# Regularized Spectral Spike Response Model: A Neuron Model for Robust Parameter Reduction

**DOI:** 10.3390/brainsci12081008

**Published:** 2022-07-29

**Authors:** Yinuo Zeng, Wendi Bao, Liying Tao, Die Hu, Zonglin Yang, Liren Yang, Delong Shang

**Affiliations:** 1 Nanjing Institute of Intelligent Technology, Nanjing 210000, China; zyn@niit.ac.cn (Y.Z.); bwd@niit.ac.cn (W.B.); hd@niit.ac.cn (D.H.); yzl@niit.ac.cn (Z.Y.); ylr@niit.ac.cn (L.Y.); 2 Institute of Microelectronics of the Chinese Academy of Sciences, Beijing 100000, China; taoliying@ime.ac.cn; 3 University of Chinese Academy of Sciences, Beijing 100000, China

**Keywords:** spike response model, neuron models, Izhikevich neuron model

## Abstract

The modeling procedure of current biological neuron models is hindered by either hyperparameter optimization or overparameterization, which limits their application to a variety of biologically realistic tasks. This article proposes a novel neuron model called the Regularized Spectral Spike Response Model (RSSRM) to address these issues. The selection of hyperparameters is avoided by the model structure and fitting strategy, while the number of parameters is constrained by regularization techniques. Twenty firing simulation experiments indicate the superiority of RSSRM. In particular, after pruning more than 99% of its parameters, RSSRM with 100 parameters achieves an RMSE of 5.632 in membrane potential prediction, a VRD of 47.219, and an F1-score of 0.95 in spike train forecasting with correct timing (±1.4 ms), which are 25%, 99%, 55%, and 24% better than the average of other neuron models with the same number of parameters in RMSE, VRD, F1-score, and correct timing, respectively. Moreover, RSSRM with 100 parameters achieves a memory use of 10 KB and a runtime of 1 ms during inference, which is more efficient than the Izhikevich model.

## 1. Introduction

Biological neurons, the fundamental building blocks of the brain, have been the subject of extensive research since the 1900s, and numerous models have been developed to describe them from various perspectives. The Hodgkin–Huxley Model [1], the Fitzhugh-Nagumo Model [2], and the Morris-Lecar Model [3] attempt to represent biophysical properties of real neurons. The Leaky Integrate-and-Fire (LIF) Model [4], the Quadratic Integrate-and-Fire Model [5], the Exponential Integrate-and-Fire Model [6], and the Izhikevich Model [7] are designed from the phenomenological viewpoint. In addition to these two categories consisting of a system of differential equations, the Point Process Model [8], the Linear-Nonlinear-Poisson (LNP) Model [9], and the Generalized Linear Model (GLM) [10] describe the input-output relation of biological neurons through a combination of a linear function representing subthreshold membrane potentials and a nonlinear function simulating spike firing rates. Although various neuron models provide valuable insights into biological neurons, it remains difficult to replicate the full functionalities of real neurons. To express the dynamics of a single biological neuron, it is necessary to implement a five-layer, fully-connected artificial neural network using deep learning technologies [11].

Either hyperparameter selection or overparameterization is cumbersome in current neuron models. First, it is challenging for neuron models to search the hyperparameter space and select the appropriate value for each hyperparameter. Since there is no effective technique to estimate hyperparameters, strategies for hyperparameter optimization depend on experts’ domain knowledge and brute-force searching algorithms, which are extremely laborious and time-consuming. In addition, the selection of hyperparameters is involved in neuron models with a system of differential equations. The Izhikevich model [7], for instance, is a two-dimensional dynamical system composed of ordinary differential equations with hyperparameters. The mechanism of neuron models is described by ordinary differential equations, whereas a set of hyperparameters correlates to a particular firing behavior. The systematic differential equation solution is computationally intensive. The iterative nature of numerical integration techniques such as the Euler’s method and the Runge–Kutta method necessitates a substantial number of iterations in order to acquire solutions. Noise makes the phenomenon more complicated, and stochastic differential equation solvers increase the computational burden even further. Second, overparameterization signifies a tremendous number of parameters. Since each parameter carries information regarding spike history, neuron models, such as the Point Process Model, invariably associate a high number of parameters [8]. Although increasing the number of parameters in a model tends to improve its accuracy, the resulting increase in computing load can impede the model’s computational efficiency.

In this article, a neuron model called the Regularized Spectral Spike Response Model (RSSRM) is proposed, which combines the Spike Response Model (SRM) [12] with the Fourier basis function. By combining their benefits, the model can overcome the aforementioned obstacles. Specifically, a data-driven algorithm for parameter estimation is implemented without hyperparameter optimization, and the enormous number of parameters is reduced by means of suitable regularization techniques.

The remaining sections of this paper are structured as follows. The Spike Response Model, the Fourier basis function, and regularization methods are reviewed in Section 2, followed by an introduction to the Regularized Spectral Spike Response Model. In Section 3, data preparation, extensive experiments, evaluation metrics, and a comprehensive model comparison are presented, while in Section 4, a discussion is provided.

## 2. Materials and Methods

This section provides an overview of the Spike Response Model, the Fourier basis function, and regularization methods. Then, the Regularized Spectral Spike Response Model, a novel neuron model, is introduced.

### 2.1. Spike Response Model

The Spike Response Model (SRM) is a neuron model that describes the input-output relation of biological neurons. It takes injected currents and spike history as inputs and produces membrane potentials. In contrast to other neuron models that are developed using differential equations, SRM is composed of linear filters and has the form

(1) $$\hat{u}\left(t\right)=u_{rest}+\int_{0}^{\infty }\kappa \left(s\right)I\left(t-s\right)ds+\sum_{f}\eta \left(t-t^{f}\right),$$

in which $\hat{u}$ is the estimated membrane potential, $u_{rest}$ is the resting potential, *I* is injected currents, κ is the filter for *I*, and $\sum_{f}\eta \left(t-t^{f}\right)$ is the filter for spike history $t^{f}$. Specifically, $\int_{0}^{\infty }\kappa \left(s\right)I\left(t-s\right)ds$ is a low-pass filter that processes the information of injected currents. For example, when the input currents are noisy, such a low-pass filter can filter out extraneous information and maintain stable, clear signals. It has been proven that linear filters are essential components for portraying the neuronal behavior in the subthreshold regime [13]. $\sum_{f}\eta \left(t-t^{f}\right)$ is a function of the spike history, which accounts for neuronal characteristics such as refractoriness after spiking.

Although linear filters form the foundation of SRM, there are strong connections between it and other neuron models that comprise a system of differential equations. First, SRM is a generalized LIF model when its analytical solution has been obtained [12]. In addition, there is a relationship between SRM and the Hodgkin–Huxley model under certain conditions [14]. Furthermore, a fast-spiking neuron model [15] can be well represented by SRM [16].

In addition to equivalent model structures between SRM and neuron models driven by a system of differential equations, the performance of SRM appears promising. A number of experiments have demonstrated that SRM is competitive in comparison to other neuron models [17].

It is crucial to select filters κ and η since they determine the performance of SRM. There are three primary methods. First, hand-crafted filters are proposed based on specific neural data. For example, the background firing rate data of cat spinal motoneurons [18] can be reproduced by SRM using filters of the following structure [12]

(2) $$\kappa \left(t-\hat{t},s\right)=\frac{R}{\tau_{m}}\;\left[1-e^{-\frac{t-\hat{t}}{\tau_{rec}}}\right]\;e^{-\frac{s}{\tau_{m}}}\;\Theta \left(S\right)\;\Theta \left(t-\hat{t}-s\right),$$

(3) $$\eta \left(t-\hat{t}\right)=-\eta_{0}\;e^{-\frac{t-\hat{t}}{\tau_{ref}}}\;\Theta \left(t-\hat{t}\right),$$

in which $\hat{t}$ indicates the last firing time. Filter $\kappa (t-\hat{t},s)$ is a modified version of filter κ(s), which takes $t-\hat{t}$ an extra input. Θ represents the Heaviside step function with Θ(s)=1 for s>0, and Θ(s)=0 else. Variables *R*, $\tau_{m}$, $\tau_{rec}$, $\tau_{m}$, $\eta_{0}$ and $\tau_{ref}$ are hyperparameters. Second, filter η can be derived from data using domain knowledge, and then filter κ can be obtained by numerical implementation. The spike-triggered average (STA) is specifically employed to extract η. Experts are able to design an appropriate function to describe η based on its shape. The filter κ can then be determined by solving the Wiener–Hopf equation [19]. Third, a linear approximation can be used to generate filters κ and η. The numerical optimization is performed to prevent manually selecting filters [20]. In this scenario, SRM has the form

(4) $$\hat{u}\left(t\right)=u_{rest}+\sum_{s=0}^{S}\kappa_{s}I_{t-s}+\sum_{q=1}^{Q}\eta_{q}S\left(t-q\right),$$

in which $S\left(t\right)=\sum_{f}\delta \left(t-t^{f}\right)$ represents spike history, *Q* is the number of time lags of S(t), and *S* is the number of time lags of injected currents *I*. A time lag conveys information pertaining to a prior or current event. For instance, membrane potentials at time t−2 and t−1 represent two time lags for the membrane potential at time *t* with lag = 2 and 1, respectively. In Equation (4), to predict membrane potential *u* at time *t*, we use the information of injected currents from time *t* to time t−S and spike history from time t−1 to time t−Q, which corresponds to *S* time lags of injected currents with lag =0,1,2,…,S and *Q* time lags of spike history with lag =1,2,…,Q.

It is difficult for SRM to solve a variety of biologically realistic tasks using the first or the second method. Since selecting filters involves hyperparameter optimization, it is arduous for specialists to develop filters that correspond to each task. The third method of applying a linear approximation for filter selection is preferred, and since it is a data-driven method, every filter can be obtained automatically from the data. This approach will be performed to fit SRM in Section 3. However, overparameterization is an issue caused by this method. SRM must memorize a large amount of information about injected currents and spike history in order to achieve a high level of performance, which dramatically increases the number of parameters and restricts its applications to biologically realistic tasks due to intensive computation and large memory occupancy.

### 2.2. Fourier Basis Function

The Fourier series is a powerful tool in various fields. In mathematics, it is an efficient alternative for solving a system of partial differential equations [21]. In deep learning, it approximates non-differentiable functions to expedite the training process [22]. In data analysis, it improves the performance of rainfall prediction models [23]. In neuroscience, it is used to investigate the dynamics of neural spike trains [24].

The Fourier series has the form

(5) $$f\left(t\right)=a_{0}+\sum_{i=1}^{\infty }\;\left[\;a_{i}cos\left(2\pi ti/T\right)+b_{i}sin\left(2\pi ti/T\right)\;\right]\;,\forall \;t=1,\ldots ,n,$$

in which *T* is the period, ${\left\{a\right\}}_{i=0}^{\infty }$ and ${\left\{b\right\}}_{i=1}^{\infty }$ are coefficients of sine and cosine components, respectively.

In a matrix form, it is represented as

(6) $$\begin{array}{cc} f(t)\; & =Fc\\ \; & =\left[\begin{array}{cccccc} 1 & cos(2\pi 1\frac{1}{n}) & sin(2\pi 1\frac{1}{n}) & cos(2\pi 2\frac{1}{n}) & sin(2\pi 2\frac{1}{n}) & \ldots \\ 1 & cos(2\pi 1\frac{2}{n}) & sin(2\pi 1\frac{2}{n}) & cos(2\pi 2\frac{2}{n}) & sin(2\pi 2\frac{2}{n}) & \ldots \\ \; & \; & & \ldots & \; & \\ 1 & cos(2\pi 1\frac{n}{n}) & sin(2\pi 1\frac{n}{n}) & cos(2\pi 2\frac{n}{n}) & sin(2\pi 2\frac{n}{n}) & \ldots \end{array}\right]\left[\begin{array}{c} a_{0}\\ a_{1}\\ b_{1}\\ a_{2}\\ b_{2}\\ \ldots \end{array}\right], \end{array}$$

in which $t={\left[1,\ldots ,n\right]}^{T}$, F is a matrix called the Fourier basis [25], and c is a vector of coefficients.

Considering the stability of estimating coefficients, the number of columns in F should not be larger than the number of rows [26]. Specifically, if *n* is odd, then

(7) $$f\left(t\right)=a_{0}+\sum_{i=1}^{(n-1)/2}\;\left[\;a_{i}cos\left(2\pi i\frac{t}{T}\right)+b_{i}sin\left(2\pi i\frac{t}{T}\right)\;\right],$$

and

(8) $$\begin{array}{c} F={\left[\begin{array}{ccccccccc} 1 & cos(2\pi 1\frac{1}{n}) & sin(2\pi 1\frac{1}{n}) & cos(2\pi 2\frac{1}{n}) & sin(2\pi 2\frac{1}{n}) & \ldots & cos(2\pi \frac{n-1}{2}\frac{1}{n}) & sin(2\pi \frac{n-1}{2}\frac{1}{n})\\ 1 & cos(2\pi 1\frac{2}{n}) & sin(2\pi 1\frac{2}{n}) & cos(2\pi 2\frac{2}{n}) & sin(2\pi 2\frac{2}{n}) & \ldots & cos(2\pi \frac{n-1}{2}\frac{2}{n}) & sin(2\pi \frac{n-1}{2}\frac{2}{n})\\ \; & \; & & \; & & \ldots & \; & & \\ 1 & cos(2\pi 1\frac{n}{n}) & sin(2\pi 1\frac{n}{n}) & cos(2\pi 2\frac{n}{n}) & sin(2\pi 2\frac{n}{n}) & \ldots & cos(2\pi \frac{n-1}{2}\frac{n}{n}) & sin(2\pi \frac{n-1}{2}\frac{n}{n})\; \end{array}\right]}_{n\times n}. \end{array}$$

If *n* is even, then

(9) $$f\left(t\right)=a_{0}+\sum_{i=1}^{n/2-1}\;\left[\;a_{i}cos\left(2\pi i\frac{t}{T}\right)+b_{i}sin\left(2\pi i\frac{t}{T}\right)\;\right]\;+a_{\frac{n}{2}}{\left(-1\right)}^{t},$$

and

(10) $$\begin{array}{c} F={\left[\begin{array}{ccccccccc} 1 & cos(2\pi 1\frac{1}{n}) & sin(2\pi 1\frac{1}{n}) & cos(2\pi 2\frac{1}{n}) & sin(2\pi 2\frac{1}{n}) & \ldots & cos(2\pi \left(\frac{n}{2}-1\right)\frac{1}{n}) & sin(2\pi \left(\frac{n}{2}-1\right)\frac{1}{n}) & -1\\ 1 & cos(2\pi 1\frac{2}{n}) & sin(2\pi 1\frac{2}{n}) & cos(2\pi 2\frac{2}{n}) & sin(2\pi 2\frac{2}{n}) & \ldots & cos(2\pi \left(\frac{n}{2}-1\right)\frac{2}{n}) & sin(2\pi \left(\frac{n}{2}-1\right)\frac{2}{n}) & {\left(-1\right)}^{2}\\ \; & \; & & \; & & \ldots & \; & & \\ 1 & cos(2\pi 1\frac{n}{n}) & sin(2\pi 1\frac{n}{n}) & cos(2\pi 2\frac{n}{n}) & sin(2\pi 2\frac{n}{n}) & \ldots & cos(2\pi \left(\frac{n}{2}-1\right)\frac{n}{n}) & sin(2\pi \left(\frac{n}{2}-1\right)\frac{n}{n}) & {\left(-1\right)}^{n} \end{array}\right]}_{n\times n}. \end{array}$$

One of the intriguing properties of the Fourier basis is its orthogonality. Orthogonality improves the estimate of coefficients {a} and {b} because it indicates that columns in **F** are independent of each other. Additionally, when performing variable selection, it is simple to filter irrelevant columns, and retain only the essential ones.

Basis function approach, as a nonparametric regression technique, is flexible to fit data [27]. It has the form [28]

(11) $$y_{t}=\beta_{0}+\beta_{1}b_{1}\left(x_{t}\right)+\beta_{2}b_{2}\left(x_{t}\right)+\beta_{3}b_{3}\left(x_{t}\right)+\ldots +\epsilon_{t},$$

in which $y_{t}$ is the dependent variable, $x_{t}$ is the independent variable, and $b_{1}\left(\cdot \right)$, $b_{2}\left(\cdot \right)$, $b_{3}\left(\cdot \right)$, …represent basis functions, which are the transformations of $x_{t}$.

The Fourier basis function applies the Fourier basis for the basis function approach, and is described by

(12) $$y_{t}=\beta_{0}+\beta_{11}b_{11}\left(x_{t}\right)+\beta_{12}b_{12}\left(x_{t}\right)+\beta_{21}b_{21}\left(x_{t}\right)+\beta_{22}b_{22}\left(x_{t}\right)+\beta_{31}b_{31}\left(x_{t}\right)+\beta_{32}b_{32}\left(x_{t}\right)+\ldots +\epsilon_{i},$$

in which basis functions are $b_{i1}=cos\left(2\pi i\frac{t}{n}\right)x_{t}$ and $b_{i2}=sin\left(2\pi i\frac{t}{n}\right)x_{t}$.

The orthogonality of the Fourier basis is inherited by the Fourier basis function so that it has advantages in both coefficients estimation and variable selection.

### 2.3. Regularization

Regularization is a technique for shrinking the magnitude of model parameters to restrict model complexity. A complex model may capture noisy signals within a given dataset, resulting in large variance and poor performance on the other dataset. By penalizing the scale of parameters, regularization methods reduce the variation and guarantee a smooth fitted model.

Regularization is incorporated into the loss function during the model fitting procedure, and constrained parameters are acquired by numerical optimization. The loss function has the form

(13) E=error+regularization,

where the error term measures the goodness-of-fit of the model, while the regularization term aims to shrink the magnitude of parameters. Some classic regularization methods are Ridge [29], LASSO [30], Elastic Net [31], and SCAD [32].

### 2.4. Regularized Spectral Spike Response Model

The Regularized Spectral Spike Response Model (RSSRM) is a neuron model that combines the Spectral Spike Response Model (SSRM) and regularization methods, where SSRM is constructed by a combination of SRM and the Fourier basis function. Specifically, SSRM has the form

(14) $$\hat{u}\left(t\right)=u_{rest}+\sum_{j=1}^{J}w_{j}\;b_{t,j}\left[I\left(t\right)\right]+\sum_{k=1}^{K}w_{k}\;b_{t-1,k}\left[S\left(t-1\right)\right],$$

in which $\hat{u}\left(t\right)$ is the estimated membrane potential, $u_{rest}$ is the resting potential, *I* is the injected currents, and $S\left(t\right)=\sum_{f}\delta \left(t-t^{f}\right)$ is the spike history. $b_{t,i}$ is the Fourier basis function that projects input currents and spike history from the time domain to the spectral domain. Specifically, $b_{t,i}\left[x\left(t\right)\right]=cos\left(i\omega t\right)\;x\left(t\right)+sin\left(i\omega t\right)\;x\left(t\right)$, where $\omega =\frac{2\pi }{T}$ and *T* is the length of time.

Similar to the third method for selecting filters κ and η in SRM mentioned in Section 2.1, a linear approximation is performed on SSRM so that filters κ and η, referred to as parameters *w* here, are obtained by numerical optimization methods.

The loss function is

(15) $$E=\frac{1}{2}\sum_{t}\;{\left[\;u\left(t\right)-\hat{u}\left(t\right)\;\right]}^{2}+\lambda (\sum_{j=1}^{J}|w_{j}|+\sum_{k=1}^{K}|w_{k}\left|\right),$$

where the first term is the Mean Squared Error, and the second term is L1 regularization [30], which shrinks the magnitude of parameters to zero so that the number of parameters is reduced. λ, a hyperparameter, controls the degree of parameter shrinkage.

After mapping to the spectral domain, SSRM is able to exploit the potential of data, requiring significantly fewer time lags than SRM. Specifically, Section 3.2 shows that with zero time lag for the injected currents and one time lag for the spike history, SSRM outperforms SRM in membrane potential prediction and spike train forecasting under the scenario of parameter reduction.

## 3. Results

In this section, the data, including twenty classic firing behaviors of biological neurons, are generated, followed by exhaustive experiments of model fitting for membrane potentials and spike trains, respectively. Then, evaluation metrics are introduced, and comprehensive model comparisons are undertaken to evaluate the performance of each model.

### 3.1. Data

The data of twenty spiking behaviors of real neurons are generated from the Izhikevich model by altering the hyperparameter values [7,33]. It is a two-dimensional dynamic system where the first equation employs the quadratic integrate-and-fire model [20], and has the form

(16) $$\left\{\begin{array}{c} \tau_{m}\frac{dv}{dt}=\left(v-v_{rest}\right)\left(v-\theta \right)-Ru+RI\\ \tau_{u}\frac{du}{dt}=a\left(v-v_{rest}\right)-u+b\tau_{u}\sum_{f}\delta \left(t-t^{f}\right) \end{array}\right.,$$

with the after-spike resetting

(17) $$\mathrm{if}\;v=\theta_{reset},\;\;\mathrm{then}\;\left\{\begin{array}{c} t^{f}\leftarrow t\\ u\leftarrow u_{r} \end{array}\right.,$$

in which *v* is the membrane potential. *u* is the auxiliary variable. $\tau_{m}$ and $\tau_{u}$ are the membrane time constants. $v_{rest}$ is the resting potential. θ is the critical voltage for spike initiation by a short current pulse. *R* is the membrane resistance. $\sum_{f}\delta \left(t-t^{f}\right)$ is the spike history. $\theta_{reset}$ is the numerical threshold. $u_{r}$ is the resting potential for the auxiliary variable. *a* and *b* are hyperparameters.

By fitting the spike initiation dynamics of a cortical neuron, dependent parameters are estimated. The simplified form is described below [7]

(18) $$\left\{\begin{array}{c} \frac{dv}{dt}=0.04v^{2}+5v+140-u+I\\ \frac{du}{dt}=a\left(bv-u\right) \end{array}\right.,$$

with the after-spike resetting

(19) $$\mathrm{if}\;v\geq 30\;\mathrm{mV},\;\;\mathrm{then}\;\left\{\begin{array}{c} v\leftarrow c\\ u\leftarrow u+d \end{array}\right.,$$

in which *v* is the membrane potential. *u* is the auxiliary variable. a,b,c, and *d* are hyperparameters.

**Figure 1:**
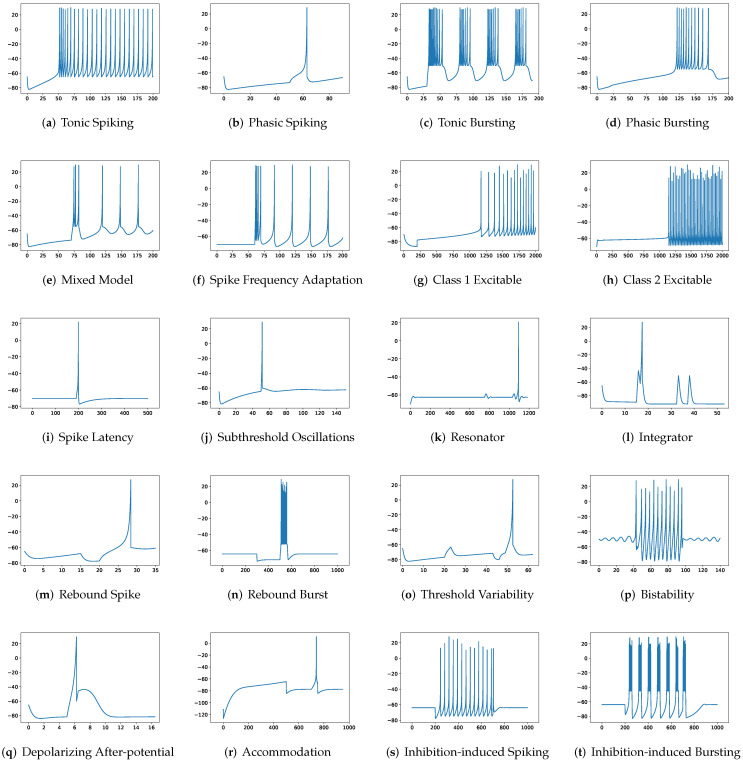
Twenty firing behaviors generated by the Izhikevich Model, where y-axis is the membrane potential (mV) and x-axis is the time (ms).

To simulate twenty spiking patterns of biological neurons, injected currents *I*, initial values of $v_{0}$ and $u_{0}$, and the value for each hyperparameter are provided. Then, the Euler’s method, a first-order numerical integration, is applied to solve the two-dimensional system of ordinary differential equations of the Izhikevich model [34]. Figure 1 shows the twenty generated spiking modes generated by the Izhikevich model, which are (a) tonic spiking, (b) phasic spiking, (c) tonic busting, (d) phasic bursting, (e) mixed mode, (f) spike frequency adaptation, (g) class 1 excitable, (h) class 2 excitable, (i) spike latency, (j) subthreshold oscillation, (k) resonator, (l) integrator, (m) rebound spike, (n) rebound burst, (o) threshold variability, (p) bistability, (q) depolarizing after-potential, (r) accommodation, (s) inhibition-induced spiking, and (t) inhibition-induced bursting.

Although the Izhikevich model strikes a balance between biological plausibility and computational efficiency [7], it is limited in its flexibility to tackle biologically realistic tasks. Given that the simplicity of the Izhikevich model is derived from the fitting of the spike initiation dynamics of a cortical neuron [7], it is unclear whether the superiority of such a modeling is universal. In addition, the Izhikevich model produces a limited number of firing patterns. Twenty firing behaviors correlate to hyperparameter values. In reality, biological neurons are diverse and sensitive to varying amplitudes of injected currents [35,36]. Consequently, the resulting spiking patterns are complicated, and it is difficult for the Izhikevich model to obtain the relevant hyperparameter values. The inability of the Izhikevich model to tackle various biologically realistic tasks is due to its restricted modeling of a single biological neuron and its hyperparameter-associated representation of firing behaviors. It is necessary to develop a novel neuron model that is adaptable enough to generate spiking behaviors that support a variety of biologically realistic tasks and that avoids the use of hyperparameters.

### 3.2. Experiments

Six neuron models, the Spike Response Model (SRM), the Regularized Spike Response Model (RSRM), the Spectral Spike Response Model (SSRM), the Regularized Spectral Spike Response Model (RSSRM), the Principal Component Regression (PCR) [28], and the Raised Cosine Basis Function Regression (RCR) [37,38,39], are applied to fit the data of twenty firing behaviors. The dependent variable, membrane potentials, is shared, whereas the independent variables are versatile. Specifically, independent variables of SRM are injected currents and spike history with different amounts of time lags. SSRM takes as inputs the Fourier basis function on injected currents and the spike history with a single time lag. RSRM is SRM with L1 regularization for the parameter reduction, while RSSRM is SSRM with L1 regularization. The features of PCR are modified inputs of SRM, where the principal component analysis, a technique for dimension reduction, is performed to transform the independent variables of SRM into the principal components. Then, the first few principal components are selected as the features of PCR to decrease the dimension. RCR takes the raised cosine basis function on injected currents and spike history with a single time lag as predictors, where the raised cosine basis function captures temporal signals close to the time of a spike with relatively few hyperparameters [39]. The details of each neuron model are shown in Table 1.

There are two aspects of the model fitting that should be emphasized. First, parameter estimation is undertaken using the same numerical optimization method. The curse of dimensionality results from the fact that some models contain more independent variables than observations, resulting in high-dimensional input data. To fairly compare all neuron models, the L2 norm with the hyperparameter λ=0.01 is performed in the optimization process to reduce the variability in the parameter estimation. Second, since the goal is to assess the effect of parameter reduction on neuron models, instead of splitting data of spiking patterns into training and test sets, the entire data is used for the model fitting, and the generalizability of models is not considered here.

In addition to prominent membrane potential prediction, excellent spike train forecasting is expected for neuron models. However, the relationship between estimated membrane potentials and predicted spike trains are unobvious. It is possible that undesirable estimated membrane potentials are from the prediction in the subthreshold regime, while estimated spike trains are outstanding. Three algorithms are proposed to convert estimated membrane potentials to estimated spike trains with a reasonable tolerance for mismatches between the actual and estimated spike trains.

Before discussing each algorithm, the following notations are introduced. The lowercase letters represent scalars, while the bold ones indicate vectors. In addition, calligraphic uppercase letters signify sets.

Algorithm 1 aims to convert membrane potentials to spike trains. The quantile *q* in line 1 is defined as a threshold value in predicted membrane potentials, where the estimated firing ratio r×β is larger than the true firing proportion *r* to take fluctuations in predicted membrane potentials into account. For example, given the true firing proportion r=10% and an inflation coefficient β=1.5, the estimated firing ratio is r×β=15%. The 85th quantile *q* indicates that 15% of predicted membrane potentials are greater than *q*. At the time *t*, if the value of the predicted membrane potential is over the threshold point *q* and is also a local maximum, then a predicted spike is established, which is formalized by lines 2 to 6.
**Algorithm 1:** Convert membrane potentials to spike trains.
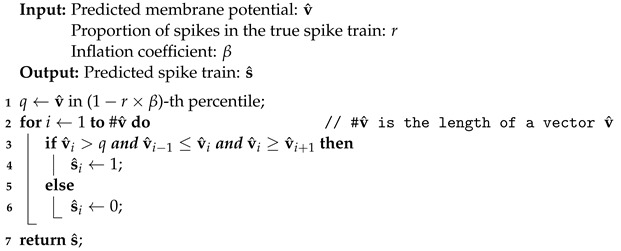


The hyperparameter β ranges from 1 to 1.5 for SRM and SSRM, and from 1 to 270 for RSRM, RSSRM, PCR, and RCR due to their significant oscillations after parameter reduction. It is introduced because the predicted membrane potentials may be subject to substantial fluctuations so that it is difficult to differentiate between these membrane potentials and potential membrane potentials for spiking. By tuning β, potential membrane potentials for spiking are accounted for, despite the inclusion of membrane potentials with substantial fluctuations. Evaluation metrics such as F1-score is applied to to accommodate this phenomenon in Section 3.3. Overall, the hyperparameter β is applied to capture all potential membrane potentials for spiking, despite the fact that some membrane potentials exhibit variations, which will be reflected in the evaluation metrics.

Algorithm 2 searches for the maximum tolerance of predicted spike trains, where maximum tolerance is a reasonable value such that predicted spikes within it are treated as the true spikes. The concept is introduced since it is common for the true spike time and the predicted spike time to differ by a few milliseconds [19]. The maximum tolerance is an auxiliary variable that improves the assessment of neuron model performance. Since the performance of each neuron model in twenty firing behaviors differs, the associated maximum tolerances can vary considerably. For a certain evaluation metric, the greater the performance, the smaller the maximum tolerance. It is straightforward to transform the output vector of spike trains from Algorithm 1 into the input set of the spike times for Algorithm 2 due to the one-to-one relationship between spike trains consisting of 0 s and 1 s and spike times denoting the precise time of firings. $\tau_{max}$ in line 1 is the upper bound of the maximum tolerance. For example, $\hat{T}=\left\{1,10\right\}$ and T={3,15}, then $\tau_{max}=\max\left(\left(10-3\right),\left(15-1\right)\right)=14$.

Within iterations of the tolerance τ, a comparison is performed between the true spike time and the predicted spike time. When all elements in either set have been enumerated, indicating that the comparison is complete, the procedure terminates and further increments of τ have no effect. Such a τ is known as the maximum tolerance. To simulate specific firing behaviors, neuron models PCR and RCR may require large values of the maximum tolerance. Since an enormous maximum tolerance is not biologically reasonable and is inefficient in applications, the value of maximum tolerance for PCR and RCR is set to 30 ms by observing that the most considerable maximum tolerance among neuron models SRM, SSRM, RSRM, and RSSRM in twenty firing behaviors is around 30 ms. These pre-defined maximum tolerances are marked by asterisks in Table 2.
**Algorithm 2:** Find maximum tolerance.
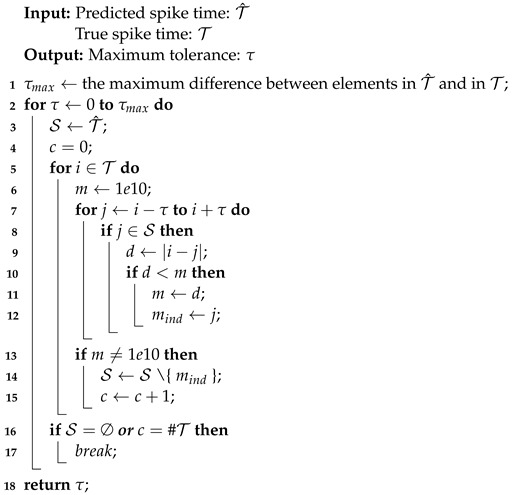


Algorithm 3 takes two sets of the spike time and a maximum tolerance as inputs and returns a predicted spike train with the maximum tolerance. This algorithm is similar to Algorithm 2, with the exception that when a predicted spike time falls inside the maximum tolerance, the true spike time is substituted.
**Algorithm 3:** Predicted spike train with maximum tolerance.
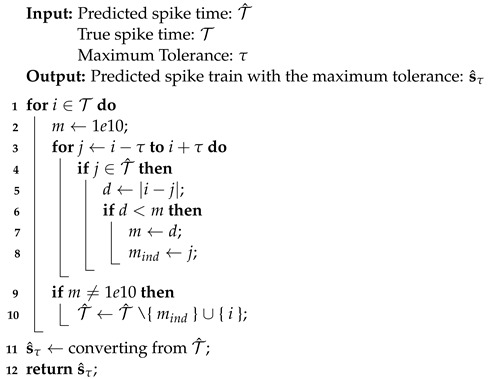


### 3.3. Evaluation

Neuron models are evaluated based on the evaluation metrics Root Mean Square Error (RMSE) [20], van Rossum distance (VRD) [40], and F1-score [41] after fixing the number of parameters. RMSE, the measure of membrane potentials, calculates the deviation between true membrane potentials derived from data of firing behaviors and predicted membrane potentials obtained from neuron models. It is defined as

(20) $$\mathrm{RMSE}=\sqrt{\frac{1}{n}\sum_{i=1}^{n}{\left(u_{i}-{\hat{u}}_{i}\right)}^{2}},$$

in which $u_{i}$ is the true membrane potential at time *i*, ${\hat{u}}_{i}$ is the predicted membrane potential at time *i*, and *n* is total time steps in the simulation.

VRD measures the similarity of two spike trains, which calculates the Euclidean distance of modified spike trains with an exponential function. It has the explicit form [42]

(21) $$\mathrm{VRD}=\sum_{i}\sum_{j}e^{-|u_{i}-u_{j}|/\tau }+\sum_{i}\sum_{j}e^{-|v_{i}-v_{j}|/\tau }-2\sum_{i}\sum_{j}e^{-|u_{i}-v_{j}|/\tau },$$

where *u* is the true spike train and *v* is the predicted spike train. Without loss of generality, the hyperparameter τ is set to be 1 for simpler calculation.

Since VRD compares a specific time of a spike train with all times of another (the third term in Equation (21)), it is unnecessary to use maximum tolerance mentioned in Algorithms 2 and 3, and the predicted spike train is obtained by Algorithm 1. Neuron models with smaller VRD are desirable.

F1-score, the measure of spike trains, computes the harmonic mean between precision and recall. Since predicted spike trains are converted from predicted membrane potentials by Algorithms 1–3, neuron models with higher F1-score and smaller maximum tolerance are preferred. F1-score has the form

(22) $$F_{1}=2\times \frac{\mathrm{precision}\times \mathrm{recall}}{\mathrm{precision}+\mathrm{recall}},$$

in which precision is the proportion of predicted spikes that are true spikes, while recall indicates the proportion of true spikes predicted by neuron models correctly. They are described below

(23) $$\mathrm{precision}=\frac{TP}{TP+FP},$$

(24) $$\mathrm{recall}=\frac{TP}{TP+FN},$$

where TP represents the True Positive that there is a fired spike and the prediction of the neuron model is correct. FP is the False Positive, indicating there is no spike but the neuron model fires. FN, the False Negative, represents a spike generated by the biological neuron, but the neuron model does not fire.

### 3.4. Model Comparison

The model comparison of neuron models is shown in Table 3. The Izhikevich model composed of a two-dimensional dynamical system of differential equations is regarded as the ground truth, where parameters are fitted by the cortical neuron data and hyperparameters are specified. The Spike Response Model (SRM) owns 9895 parameters on average of twenty firing behaviors. In comparison, the Spectral Spike Response Model (SSRM) has an average of 29,659 parameters, which is a result of applying the Fourier basis function to injected currents and spike histories, respectively. In order to acquire 100 parameters on average in the Regularized Spike Response Model (RSRM), a parameter reduction, pruning more than 99% of parameters, is performed on SRM using L1 regularization for each firing behavior. To fairly compare different neuron models, the number of parameters for the Regularized Spectral Spike Response Model (RSSRM), the Principal Component Regression (PCR), and the Raised Cosine Basis Function Regression (RCR) is restricted and is the same as in RSRM for each firing pattern. Details are shown in Table 2. Specifically, RSSRM applies a similar procedure to RSRM, where L1 regularization is conducted on SSRM in order to control the number of parameters in RSSRM to be the same as in RSRM by selecting hyperparameters appropriately. PCR utilizes the first *n* principal components as new features [28], where *n* is the number of parameters in RSRM. The corresponding *n* parameters are achieved by numerical optimization. Due to the fact that the dimensionality of the raised cosine basis is a hyperparameter, the required parameters in RCR are controlled.

**Figure 2:**
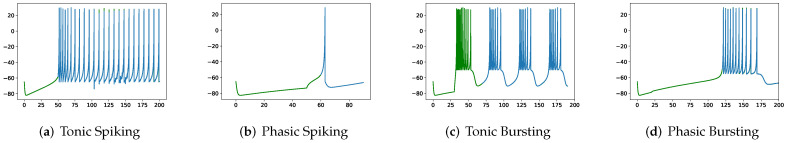

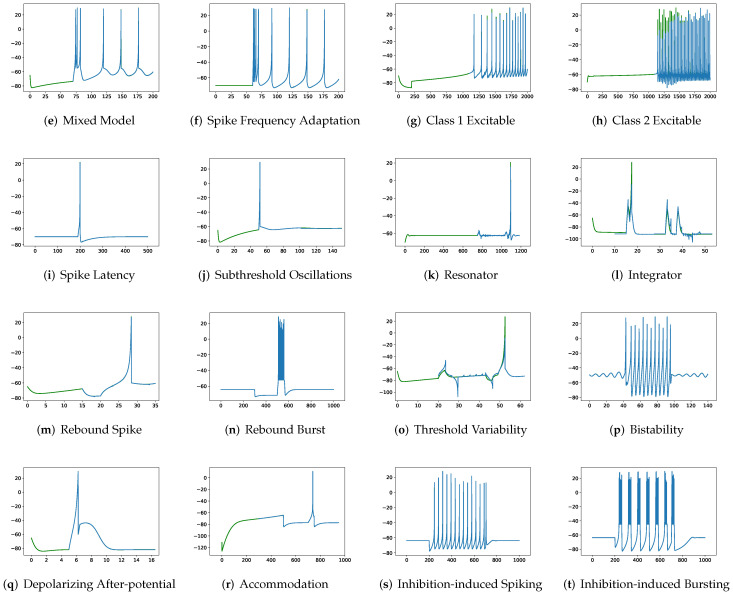
Membrane potentials of SRM (blue) vs. Izhikevich Model (green), where y-axis is the membrane potential (mV) and x-axis is the time (ms).

**Figure 3:**
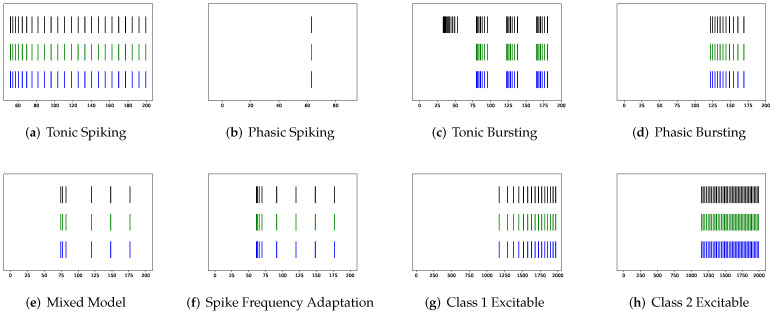

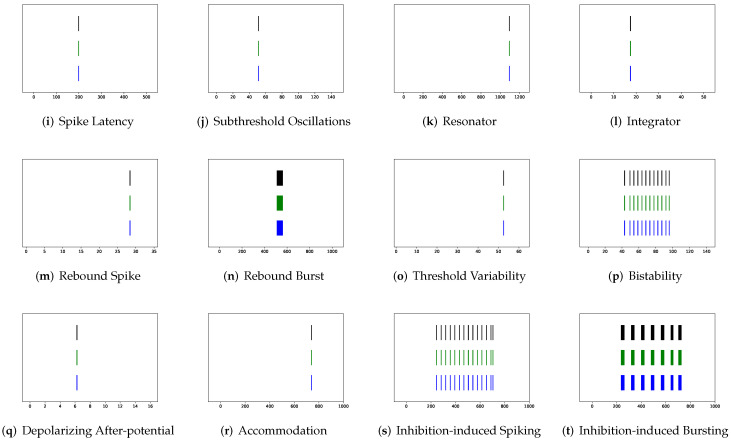
Spike trains of Izhikevich Model (black) vs. unmodified SRM (green) vs. modified SRM by maximum tolerance (blue), where x-axis is the time (ms).

**Figure 4:**
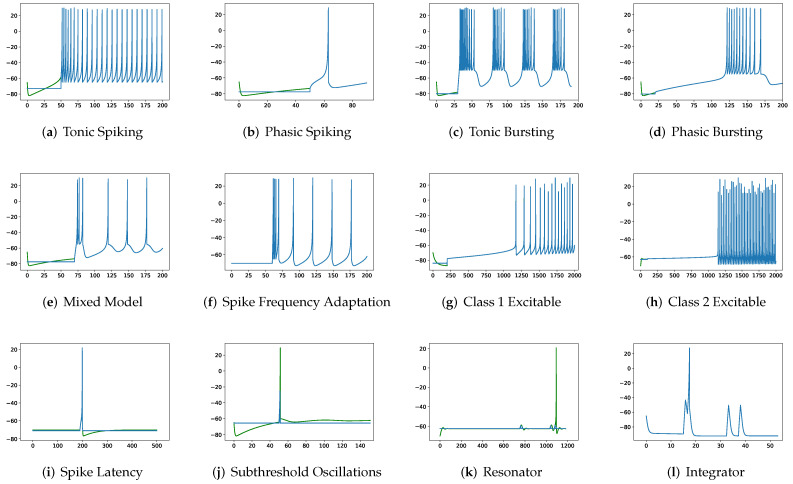

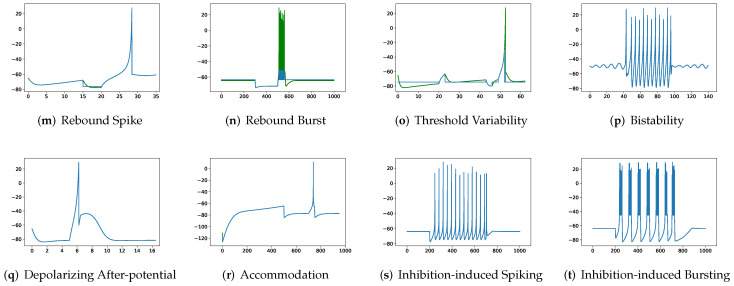
Membrane potentials of SSRM (blue) vs. Izhikevich Model (green), where y-axis is the membrane potential (mV) and x-axis is the time (ms).

**Figure 5:**
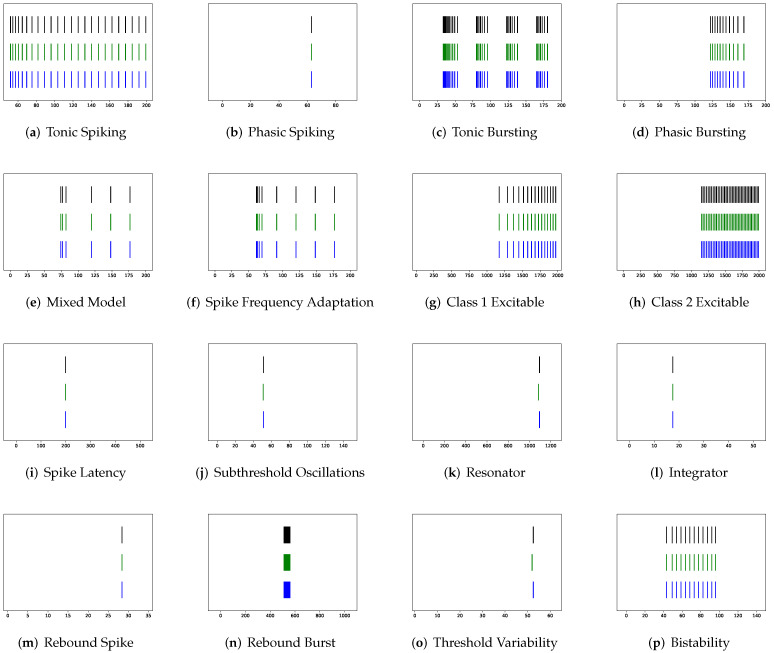

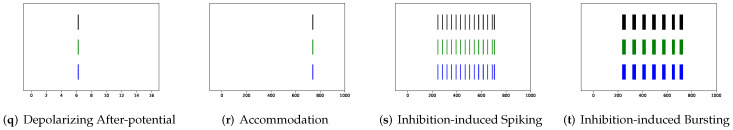
Spike trains of Izhikevich Model (black) vs. unmodified SSRM (green) vs. modified SSRM by maximum tolerance (blue), where x-axis is the time (ms).

Although SRM has significantly fewer parameters than SSRM, its overall performance is superior. In particular, SRM it has a faster inference time, lower RMSE, and smaller maximum tolerance than SSRM, as well as comparable VRD and F1-score despite requiring slightly larger memory for inference, i.e., simulating firing behaviors. Both SRM and SSRM are well performed on the vast majority of firing behaviors, which are shown in Figure 2, Figure 3, Figure 4 and Figure 5. However, the parameter reduction of SRM is not robust. After pruning 99% of parameters using L1 regularization, the inference memory, RMSE, VRD, F1-score, and maximum tolerance of RSRM are inferior than those of RSSRM with the same number of parameters. Specifically, inference memory, RMSE and maximum tolerance of RSSRM are about half as tiny as those of RSRM, while VRD and F1-score of RSSRM are 72,000 times less and 8 times higher than those of RSRM, respectively. Their difference in inference time is minor, as both require approximately 1 ms, which is far faster than the Izhikevich model.

In addition, the robustness of parameter reduction in RSSRM has been demonstrated by comparing the changes in F1-score before and after the parameter pruning. RSSRM’s F1-score decreases by only 0.05 after parameter reduction, while F1-score of RSRM is reduced drastically. Furthermore, the difference in RMSE and VRD between RSSRM and SSRM is trivial when compared to the difference between RSRM and SRM. Specifically, the RMSE of RSSRM and RSRM is approximately 5 and 20 times larger than that of SSRM and SRM, whereas the VRD of RSSRM and RSRM is 47 and 3,600,000 times larger, respectively. Moreover, their changes in inference memory and inference time are noteworthy. After parameter pruning, the memory usage and runtime of RSSRM during inference are 56 times lower and 116 times faster than those of SSRM, although RSRM has 41 times larger memory and 72 times faster runtime than SRM.

Although PCR and RCR have superior overall performance than RSRM, they are not able to compete with RSSRM, which has the lowest RMSE, the lowest VRD, the highest F1-score, and the smallest maximum tolerance among these four neuron models with parameter reduction. The inadequate performance of PCR is further demonstrated by the low variance explanation. On average for twenty firing behaviors, the first 100 principal components of PCR explain only about 51% of the variance in data. In an ideal scenario, the first few principal components should explain more than 80% of variance. Since PCR assumes that principal components are obtained by a linear combination of the original data, non-linear methods are advocated. Therefore, RSSRM and RCR, obtaining nonlinearity from basis functions, perform better than PCR. RSSRM and RCR achieve the best and the second-best performance among neuron models with parameter reduction. However, considering the involvement of hyperparameters in constructing the raised cosine basis in RCR, RSSRM, consisting of the hyperparameter-free Fourier basis function, is preferable.

In addition to evaluating the performance of neuron models on an average of twenty firing behaviors, their capacity on different categories of spiking modes is studied, respectively. Twenty spiking patterns are classified into two types based on the number of spikes. One class, as shown in Table 4, is the one-spike firing behavior, which includes (b) phasic spiking, (i) spike latency, (j) subthreshold oscillations, (k) resonator, (i) integrator, (m) rebound spike, (o) threshold variability, (q) depolarizing after-potential, and (r) accommodation. These nine spiking modes each produce a single spike throughout the experiment. As illustrated in Table 5, the other class contains eleven spiking patterns that fire multiple spikes during the simulation. It consists of (a) tonic spiking, (c) tonic bursting, (d) phasic bursting, (e) mixed mode, (f) spike frequency adaptation, (g) class 1 excitable, (h) class 2 excitable, (n) rebound burst, (p) bistability, (s) inhibition-induced spiking, and (t) inhibition-induced bursting. Table 4 demonstrates that SRM is superior to SSRM in terms of the number of parameters, inference time, RMSE, and maximum tolerance. After parameter reduction, RSSRM beats RSRM in terms of inference memory, RMSE, VRD, and F1-score. With only 50 parameters, PCR has the best performance among the neuron models with parameter reduction. It has the fastest inference time, the smallest RMSE, the lowest VRD, the highest F1-score, and the smallest maximum tolerance. Although its memory usage during inference is greater than that of other neuron models with parameter reduction, it is significantly lower than that of the Izhikevich model. PCR is the most notable model for predicting one-spike firing behaviors, whereas RSSRM is the second-best model. In Table 5, it is challenging to distinguish between the performance of SRM and SSRM. Although SRM has fewer parameters, a faster inference time, and a smaller RMSE, SSRM shows lower memory usage, a lower VRD, a higher F1-score, and a smaller maximum tolerance. When parameter reduction is considered, RSSRM has the lowest memory consumption, the lowest RMSE, the lowest VRD, the highest F1-score, and the smallest maximum tolerance among all neuron models. Furthermore, RCR is the second most prominent neuron model though the maximum tolerance is greater than twelve times that of RSSRM. Moreover, RSRM is the most unsatisfactory neuron model. Compared with RSSRM, it has around twice the RMSE, more than seventy thousand times the VRD, one-ninth the F1-score, and more than seven times the maximum tolerance. Comparing Table 4 and Table 5, the Izhikevich model doubles the memory usage for inference from one-spike firing behaviors to multiple-spike firing patterns, but the other neuron models do not exhibit this increase. Moreover, RSSRM demonstrates a consistent inference time, whereas the inference time for the other neuron models increases dramatically.

Although Table 3, Table 4 and Table 5 demonstrate that the overall performance of RSSRM is prominent among neuron models with parameter reduction, it is worthwhile to measure the capability of neuron models for each firing behavior. Details are shown in Table 2.

First, SRM, containing fewer parameters, exhibits faster inference time than SSRM in twenty firing patterns with the exception of (b) phasic spiking. Furthermore, RMSE of SRM for spiking behaviors (i) spike latency, (j) subthreshold oscillations, (k) resonator, (m) rebound spike, (n) rebound burst, and (o) threshold variability is smaller than that of SSRM. However, VRD and F1-score of both SRM and SSRM are nearly all 0.0 and 1.0 for twenty firing behaviors, respectively, indicating that perfect spike train prediction, the maximum tolerance of SSRM for (j) subthreshold oscillations, (k) resonator, (n) Rebound Burst, and (o) threshold variability is substantially greater than those of SRM, which remains zero, indicating perfect matches between the predicted and true spike trains. Zero maximum tolerance for SSRM is shown for (i) spike latency and (m) rebound spike due to a moderate corresponding RMSE.

**Figure 6:**
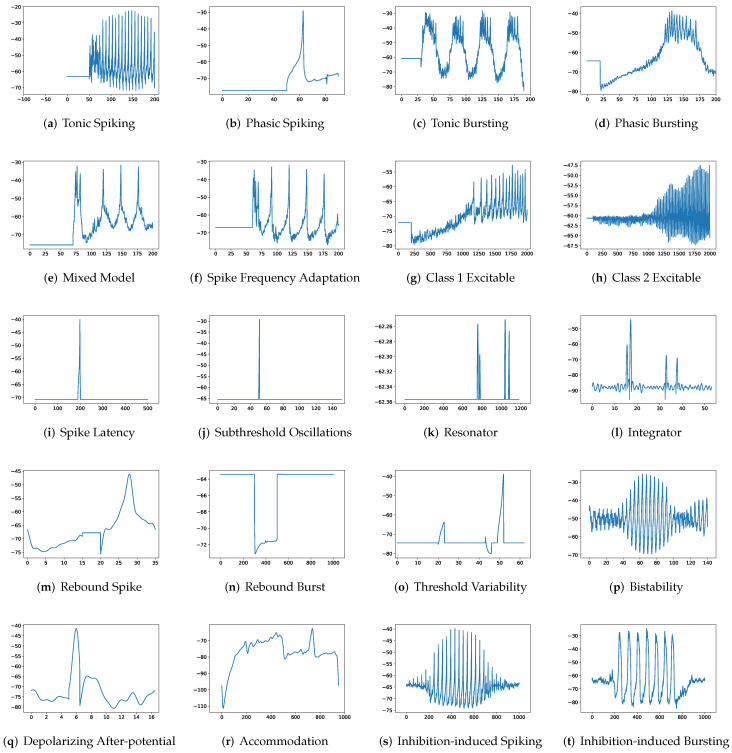
Membrane potentials of RSSRM, where y-axis is the membrane potential (mV) and x-axis is the time (ms).

In addition, in contradiction to the other firing behaviors, RMSE of RSSRM for (j) subthreshold oscillations and (n) rebound burst are larger than those of RSSRM. Specifically, the comparable RMSE of SSRM and RSSRM for (j) subthreshold oscillations indicates the Fourier basis function cannot capture the variability of membrane potentials, as depicted in Figure 4 and Figure 6. When it comes to (n) rebound burst, Figure 4 and Figure 6 reveal that neither SSRM nor RSSRM are able to reproduce the membrane potentials during spikes firing precisely.

**Figure 7:**
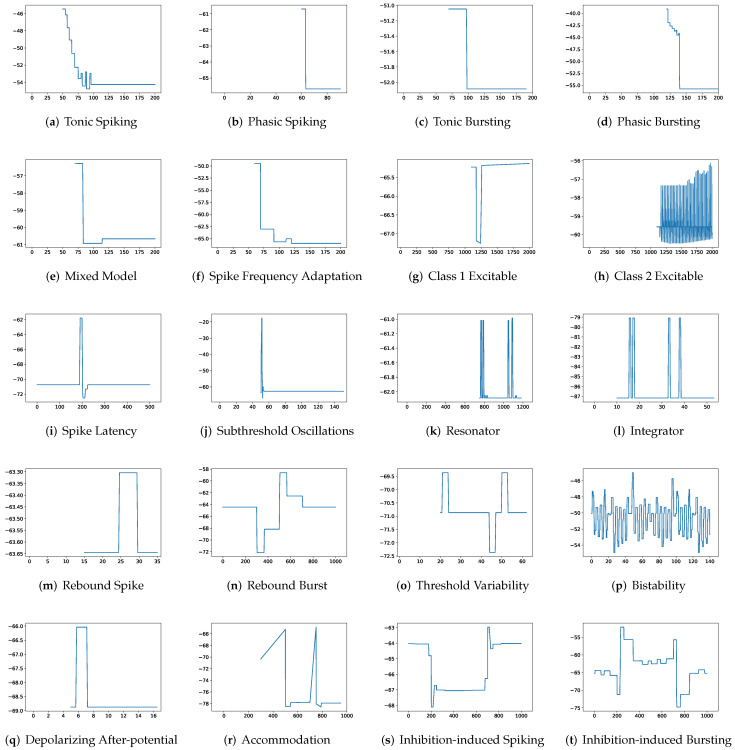
Membrane potentials of RSRM, where y-axis is the membrane potential (mV) and x-axis is the time (ms).

**Figure 8:**
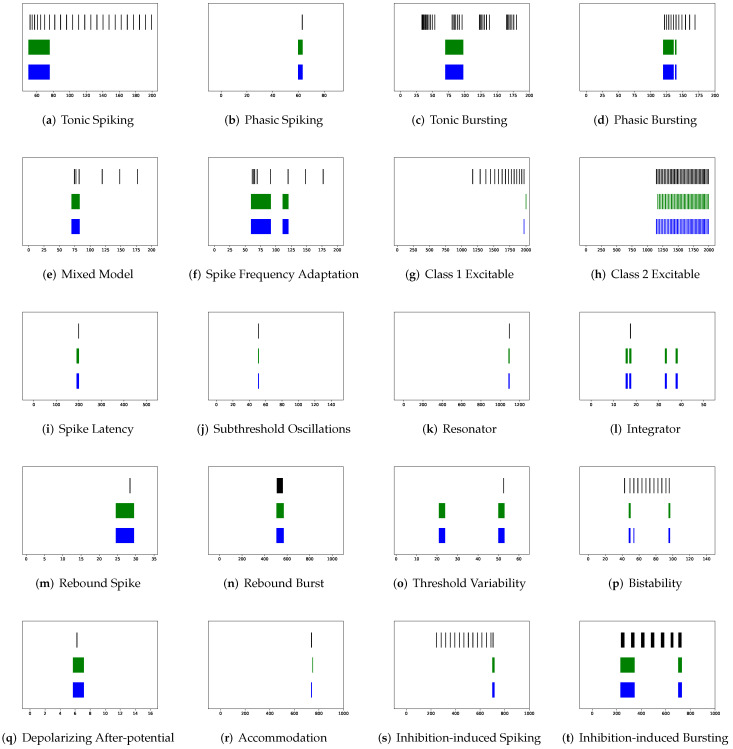
Spike trains of Izhikevich model (black) vs. unmodified RSRM (green) vs. modified RSRM by maximum tolerance (blue), where x-axis is the time (ms).

Furthermore, Table 2 show that none of RSRM owns a higher F1-score and a lower VRD than RSSRM though maximum tolerance of RSRM for (a) tonic spiking, (b) phasic spiking, (f) spike frequency adaptation, (i) spike latency, (j) subthreshold oscillations, and (k) resonator are all zeros. The rationale is indicated in Figure 7 and Figure 8, where RSRM is incapable of reproducing those firing behaviors precisely and instead provides a combination of step functions. In contrast to this circumstance, there are cases in which a considerable tolerance is required. To capture firing behaviors (g) class 1 excitable, (h) class 2 excitable, (r) accommodation, and (s) inhibition-induced spiking, the maximum tolerance values for RSRM range from 10.3 to 30.3 ms.

Moreover, Table 2 illustrates that, among all neuron models, RSSRM takes the lowest memory consumption during inference, whereas the Izhikevich model shows the highest memory usage for twenty spiking patterns, excluding (k) resonator, (m) rebound spike, (o) threshold variability and (q) depolarizing after-potential. Compared to SRM, SSRM requires smaller memory for twenty firing behaviors despite a slower inference time. Specifically, the memory consumption of SSRM is lower for fifteen firing behaviors (b) phasic spiking, (c) tonic bursting, (e) mixed mode, (f) spike frequency adaptation, (g) class 1 excitable, (h) class 2 excitable, (i) spike latency, (k) resonator, (m) rebound spike, (n) rebound burst, (o) threshold variability, (p) bistability, (q) depolarizing after-potential, (r) accommodation, and (t) inhibition-induced bursting, while SSRM has only one faster inference time than SRM for (b) phasic spiking. Among neuron models with parameter reduction, RSRM has the second-lowest memory usage in twenty firing patterns. Although PCR shows the highest memory consumption in nine spiking modes, it consumes the largest memory on average, as shown in Table 3. A comparison of inference time among neuron models with parameter reduction is trivial, as all of them takes about 1 ms during inference, which is over thirty times faster than the Izhikevich model.

To compare neuron models with parameter reduction, Table 6 ranks the performance of RSRM, RSSRM, PCR, and RCR across twenty firing behaviors. RSSRM achieves the smallest RMSE for eleven spiking patterns, including (a) tonic spiking, (b) phasic spiking, (c) tonic bursting, (d) phasic bursting, (f) spike frequency adaptation, (g) class 1 excitable, (h) class 2 excitable, (i) spike latency, (p) bistability, (s) inhibition-induced spiking, and (t) inhibition-induced bursting, and the second-smallest RMSE for five firing modes, which are (e) mixed mode, (k) resonator, (l) integrator, (m) rebound spike, and (r) accommodation. Specifically, the difference between RSSRM and the best neuron model in terms of RMSE is less than 0.5. RSSRM produces the third-smallest RMSE among the other four spike behaviors, and the difference between it and the second-best model is minor, except for (q) depolarizing after-potential, where the difference is around 0.6.

Taking spike train forecasting into account, RSSRM achieves the highest F1-score for ten firing modes containing (a) tonic spiking, (b) phasic spiking, (c) tonic bursting, (d) phasic bursting, (e) mixed mode, (f) spike frequency adaptation, (h) class 2 excitable, (i) spike latency, (n) rebound burst, and (p) bistability, and the second-highest F1-score for six spiking patterns, which includes (g) class 1 excitable, (k) resonator, (m) rebound spike, (q) depolarizing after-potential, (s) inhibition-induced spiking, and (t) inhibition-induced bursting. Specifically, RSSRM maintains a perfect F1-score for (g) class 1 excitable, (m) rebound spike, and (q) depolarizing after-potential despite a slightly larger maximum tolerance. RSSRM obtains the third-highest F1-score among the other four firing modes. While the perfect F1-score remains, the corresponding maximum tolerance is insufficient. Moreover, RSSRM accomplishes the lowest VRD across twenty firing behaviors, with the exception of (d) phasic bursting, (k) resonator, (s) inhibition-induced spiking, and (t) inhibition-induced bursting, where the second-lowest VRD is obtained. Although the rankings of VRD and F1-score for RSSRM fluctuate across eight firing modes, they are nearly identical when the influence of maximum tolerance is removed.

Moreover, PCR obtains six of the smallest RMSE, nine of the lowest VRD, and six of the highest F1-score among twenty spiking behaviors, whereas RCR achieves two of the smallest RMSE, twelve of the lowest VRD, and six of the highest F1-score. Specifically, RSSRM and RCR earn the highest F1-score for (n) rebound burst, while PCR and RCR accomplish the highest F1-score for (l) integrator. Although RSRM does not attain the highest F1-score for any firing behavior, it achieves the smallest RMSE for (j) subthreshold oscillations though the corresponding F1-score is the lowest.

In Table 6, RSSRM achieves the smallest RMSE, the lowest VRD, and the highest F1-score for eleven, sixteen, and ten firing behaviors, respectively, outperforming the other neuron models with parameter reduction.

**Figure 9:**
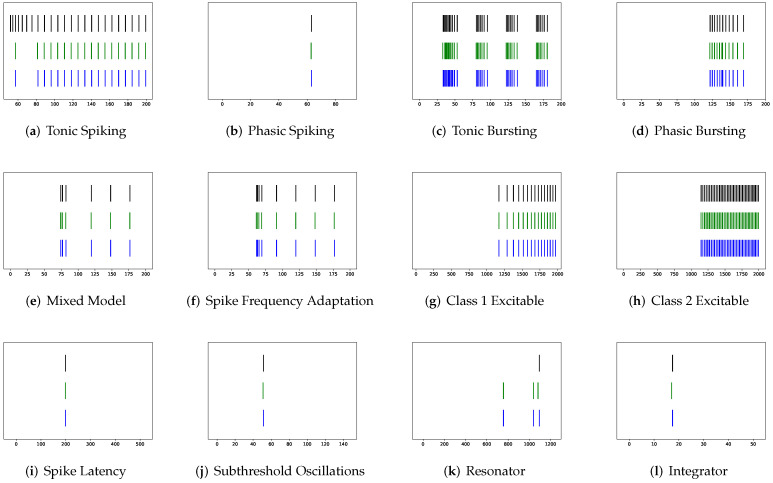

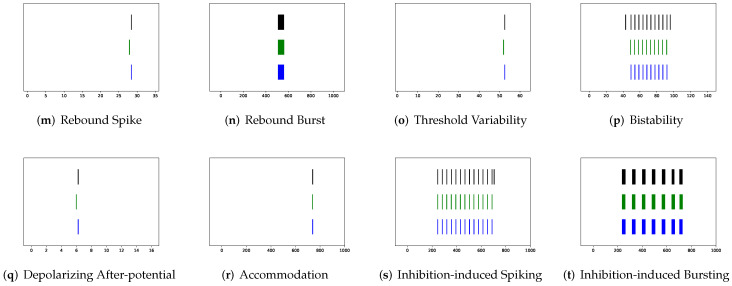
Spike trains of Izhikevich Model (black) vs. unmodified RSSRM (green) vs. modified RSSRM by maximum tolerance (blue), where x-axis is the time (ms).

**Figure 10:**
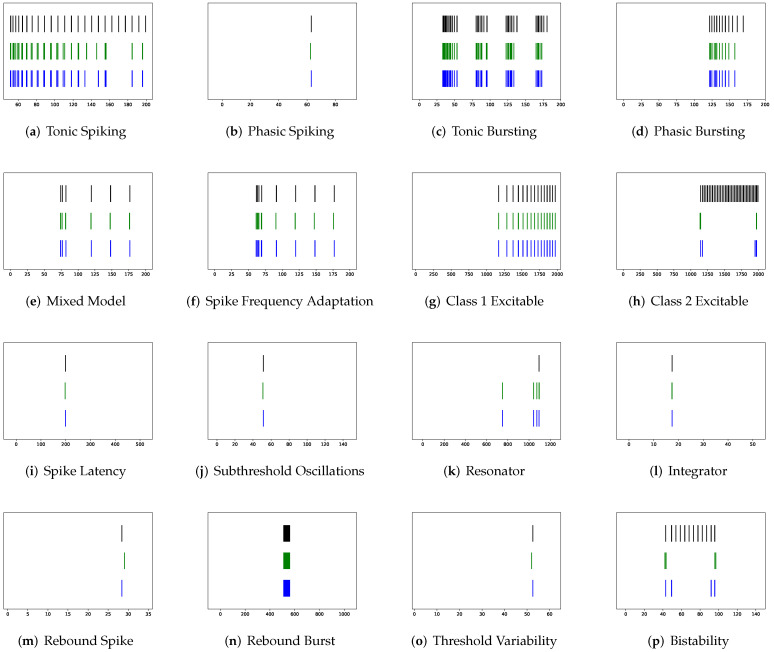

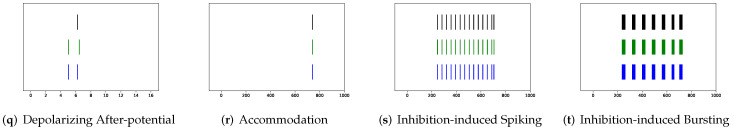
Spike trains of Izhikevich model (black) vs. unmodified RCR (green) vs. modified RCR by maximum tolerance (blue), where x-axis is the time (ms).

**Figure 11:**
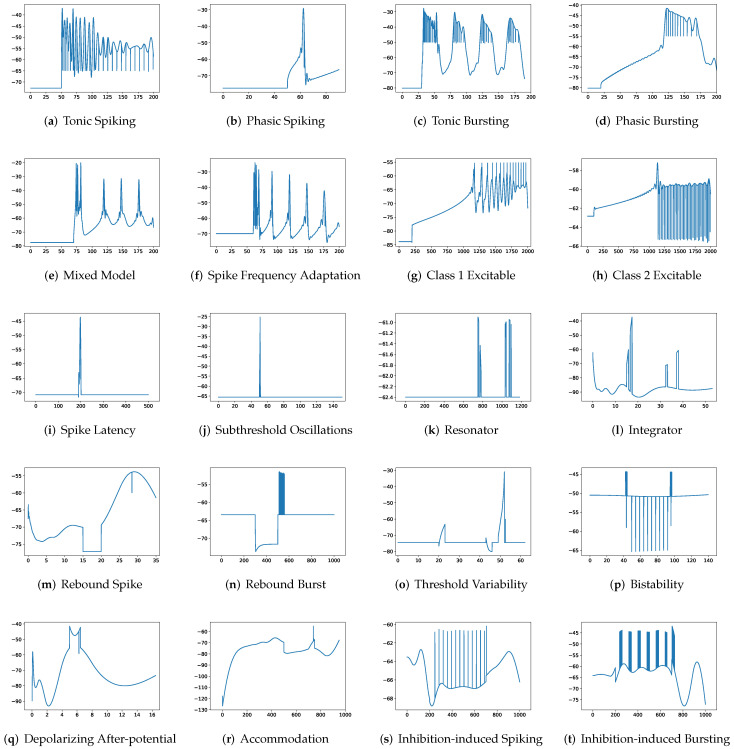
Membrane potentials of RCR, where y-axis is the membrane potential (mV) and x-axis is the time (ms).

**Figure 12:**
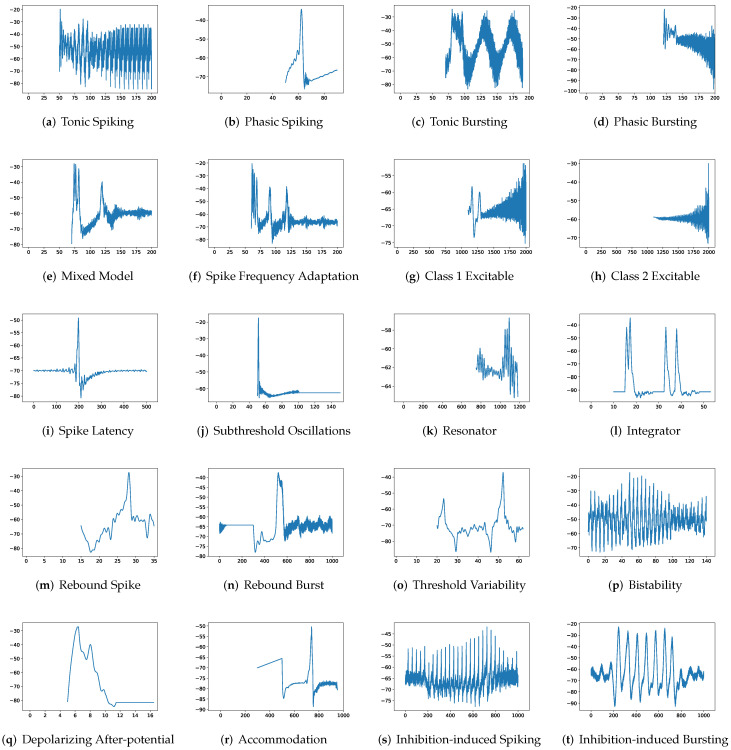
Membrane potentials of PCR, where y-axis is the membrane potential (mV) and x-axis is the time (ms).

**Figure 13:**
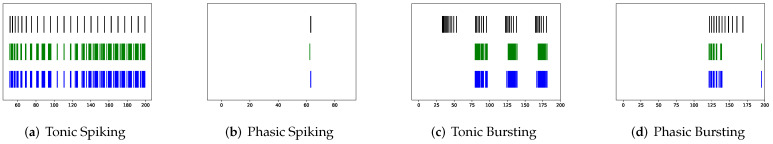

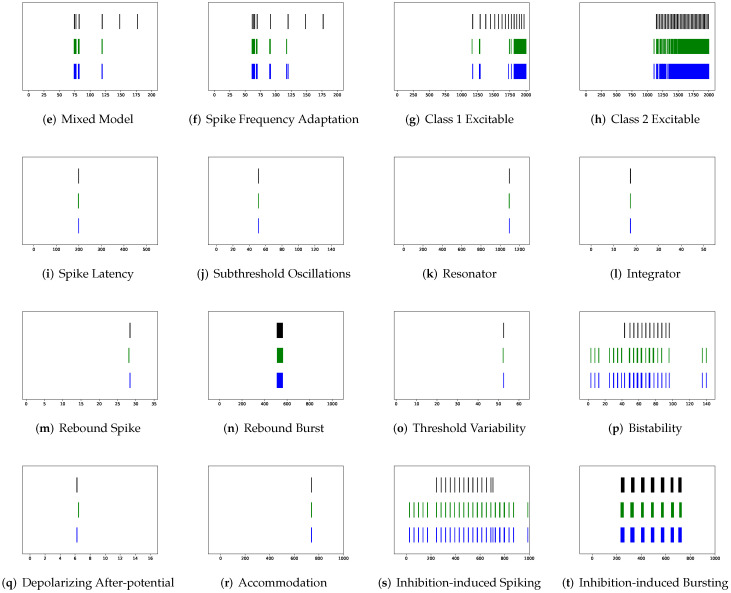
Spike trains of Izhikevich Model (black) vs. unmodified PCR (green) vs. modified PCR by maximum tolerance (blue), where x-axis is the time (ms).

Corresponding rankings support such an observation, where RMSE, VRD, and F1-score rankings of RSSRM are 1.65, 1.2, and 1.7, respectively. The superior performance of RSSRM is attributed to its excellent modeling strategy. Under the Fourier basis function, one time lag is imposed so that the model takes advantage of data maximally, which are shown in Figure 6 and Figure 9. Constructing RSRM requires an enormous number of time lags, which wastes a substantial quantity of data. Table 1, Figure 7 and Figure 8 present this phenomenon. With the second-lowest VRD and the second-highest F1-score, RCR, endows the potential in spike train prediction at the expense of membrane potential forecasting. Figure 10 shows the prominent spike train prediction, whereas Figure 11 demonstrates that predicted membrane potentials in firing behaviors such as (c) tonic bursting, (d) phasic bursting, (h) class 2 excitable, and (p) bistability are dramatically different from those of other neuron models, which use abnormal downward vertical lines to represent spikes. The performance of PCR is extreme, since the rankings of RMSE, VRD, and F1-score for the majority of firing patterns are either first or third. Figure 12 and Figure 13 illustrate that substantial fluctuations in the subthreshold regime contribute to its undesirable RMSE. Nevertheless, its performance exceeds expectations, considering that the modeling does not include the temporal structure.

## 4. Discussion

In this article, in order to overcome the challenges of hyperparameter optimization and overparameterization, which impede the deployment of neuron models in different biologically realistic tasks, the Regularized Spectral Spike Response Model (RSSRM) is proposed. This model avoids hyperparameter selection by data-driven methods and solves overparameterization through regularization approaches.

A comprehensive comparison of neuron models with parameter reduction is conducted, including the Regularized Spike Response Model (RSRM), the Regularized Spectral Spike Response Model (RSSRM), the Principal Component Regression (PCR), and the Raised Cosine Basis Function Regression (RCR). Their predictability of membrane potentials and spike trains is evaluated based on Root Mean Square Error (RMSE), van Rossum distance (VRD), F1-score, and maximum tolerance. On average of twenty firing behaviors, RSSRM with 100 parameters achieves the best performance, as demonstrated by the smallest RMSE (5.632), the lowest VRD (47.219), the highest F1-score (0.95), and the smallest maximum tolerance (1.4 ms). RSSRM with 141 parameters achieves the lowest RMSE (6.086), the lowest VRD (85.394), the highest F1-score (0.954), and the smallest maximum tolerance (0.997 ms) for the class of multiple-spike firing behaviors, whereas RSSRM with 50 parameters is the second-best neuron model that obtains the second-lowest RMSE (5.078), the second-lowest VRD (0.562), second-highest F1-score (0.944), and the fourth-smallest maximum tolerance (1.989 ms). RSSRM demonstrates superior performance in membrane potential prediction and spike train forecasting in the majority of spiking patterns examining twenty firing behaviors independently. After ranking the capacity of neuron models with parameter reduction for each firing behavior, RSSRM achieves the top position on average, where RMSE, VRD, and F1-score rankings are 1.65, 1.2, and 1.7, respectively. In addition, RSSRM achieves the lowest memory consumption (<10 KB) and approximate 1 ms runtime during inference among neuron models, which is 248 times lower and 25 times faster than the Izhikevich model, as shown in Table 3.

However, there are some limitations to this work. First, we only evaluate neuron models with L1 regularization and do not consider the other regularization methods such as elastic net and SCAD. Second, our simulated data of twenty firing behaviors is generated from the Izhikevich model. It is worthwhile to evaluate the performance of neuron models using data derived from biological neurons. Third, the performance of biological neural networks composing RSSRM is not evaluated since the involvement of learning rules and network structures potentially complicates the analysis. These problems will be investigated in future research.

## Figures and Tables

**Table 1:** Attributes of neuron models.

Model	Independent Variables	Transformation	Regularization
SRM	$I_{t},I_{t-1},\ldots ,I_{t-J},S_{t-1},\ldots ,S_{t-K}$	/	/
SSRM	$I_{t},S_{t-1}$	Fourier basis function	/
RSRM	$I_{t},I_{t-1},\ldots ,I_{t-J},S_{t-1},\ldots ,S_{t-K}$	/	L1
RSSRM	$I_{t},S_{t-1}$	Fourier basis function	L1
PCR	$I_{t},I_{t-1},\ldots ,I_{t-J},S_{t-1},\ldots ,S_{t-K}$	Principal component analysis	/
RCR	$I_{t},S_{t-1}$	Raised cosine basis function	/

**Table 2:** The performance on twenty firing behaviors.

	(a) Tonic Spiking					
**Model**	**No. Params**	**Inference Memory (MB)** ^1^	**Inference Time (s)**	**RMSE**	**VRD** ^2^	**F1-Score (Maximum Tolerance (ms))**
Izhikevich	/	3.42	0.034	0	0	1 (0)
SRM	10,000	0.52	0.056	0.451	1.264	0.980 (0.02)
SSRM	39,998	0.56	0.252	6.93 × 10${}^{-7}$	0	1 (0)
RSRM	95	0.05	0.0007	15.092	7.81 × 10${}^{6}$	0.006 (0)
RSSRM	95	0	0.0008	8.844	45.513	0.857 (0.55)
PCR	95	0.77	0.0006	12.850	3.92 × 10${}^{5}$	0.079 (30 *)
RCR	95	0.48	0.0008	12.472	126.424	0.621 (30 *)
	**(b) Phasic Spiking**					
**Model**	**No. Params**	**Inference Memory (MB)**	**Inference Time (s)**	**RMSE**	**VRD**	**F1-Score (Maximum Tolerance (ms))**
Izhikevich	/	3.25	0.033	0	0	1 (0)
SRM	11,000	0.77	0.056	0.084	0	1 (0)
SSRM	17,998	0	0.052	1.14 × 10${}^{-6}$	0	1 (0)
RSRM	109	0.08	0.0007	8.910	1.12 × 10${}^{5}$	0.007 (0)
RSSRM	108	0	0.0014	2.380	0	1 (0.25)
PCR	109	0.51	0.0007	7.937	0	1 (0.67)
RCR	109	1.11	0.0034	4.084	0	1 (0.56)
	**(c) Tonic Bursting**					
**Model**	**No. Params**	**Inference Memory (MB)**	**Inference Time (s)**	**RMSE**	**VRD**	**F1-Score (Maximum Tolerance (ms))**
Izhikevich	/	3.19	0.032	0	0	1 (0)
SRM	10,000	0.95	0.052	0.033	0	1 (0)
SSRM	37,998	0.59	0.225	3.85 × 10${}^{-7}$	0	1 (0)
RSRM	100	0.05	0.0012	17.184	9.17 × 10${}^{6}$	0.005 (1.5)
RSSRM	99	0	0.0009	7.828	0	1 (0.23)
PCR	100	0.95	0.0006	12.190	7.22 × 10${}^{4}$	0.135 (30 *)
RCR	100	0.31	0.0015	8.677	5.057	0.773 (30 *)
	**(d) Phasic Bursting**					
**Model**	**No. Params**	**Inference Memory (MB)**	**Inference Time (s)**	**RMSE**	**VRD**	**F1-Score (Maximum Tolerance (ms))**
Izhikevich	/	3.63	0.031	0	0	1 (0)
SRM	9832	0.83	0.0631	0.281	0	1 (0)
SSRM	39,998	0.86	0.2568	5.59 × 10${}^{-5}$	0	1 (0)
RSRM	101	0.04	0.0006	13.949	3.29 × 10${}^{6}$	0.007 (0.01)
RSSRM	100	0	0.0008	6.558	1.264	0.957 (0.43)
PCR	101	1.3	0.0004	12.623	2.41 × 10${}^{4}$	0.075 (30 *)
RCR	101	0.55	0.0008	7.324	0	0.727 (30 *)
	**(e) Mixed Mode**					
**Model**	**No. Params**	**Inference Memory (MB)**	**Inference Time (s)**	**RMSE**	**VRD**	**F1-Score (Maximum Tolerance (ms))**
Izhikevich	/	3.38	0.032	0	0	1 (0)
SRM	14,000	0.68	0.072	0.087	0	1 (0)
SSRM	39,998	0.64	0.259	3.39 × 10${}^{-7}$	0	1 (0)
RSRM	137	0	0.0007	10.016	1.92 × 10${}^{6}$	0.005 (0.33)
RSSRM	136	0	0.001	4.095	0	1 (0.26)
PCR	136	0.9	0.0007	7.331	1.07 × 10${}^{4}$	0.077 (30 *)
RCR	136	0.84	0.0013	3.969	1.264	0.923 (0.62)
	**(f) Spike Frequency Adaptation**					
**Model**	**No. Params**	**Inference Memory (MB)**	**Inference Time (s)**	**RMSE**	**VRD**	**F1-Score (Maximum Tolerance (ms))**
Izhikevich	/	3.23	0.031	0	0	1 (0)
SRM	12,000	0.87	0.057	0.054	0	1 (0)
SSRM	39,998	0.46	0.257	2.08 × 10${}^{-8}$	0	1 (0)
RSRM	127	0.05	0.0008	10.677	1.98 × 10${}^{7}$	0.003 (0)
RSSRM	127	0	0.001	5.309	0	1.000 (0.4)
PCR	127	0.88	0.0007	8.888	1.07 × 10${}^{4}$	0.111 (30 *)
RCR	127	0.95	0.001	5.486	20.228	0.8 (1.23)
	**(g) Class 1 Excitable**					
**Model**	**No. Params**	**Inference Memory (MB)**	**Inference Time (s)**	**RMSE**	**VRD**	**F1-Score (Maximum Tolerance (ms))**
Izhikevich	/	3.6	0.039	0	0	1 (0)
SRM	19,284	0.97	0.069	0.299	0	1 (0)
SSRM	39,998	0.95	0.252	1.67 × 10${}^{-7}$	0	1 (0)
RSRM	200	0.06	0.0011	8.010	247.791	0.125 (30.3)
RSSRM	199	0	0.0014	3.919	0	1 (1.4)
PCR	200	1.28	0.0007	7.674	2.92 × 10${}^{4}$	0.099 (30 *)
RCR	200	1.28	0.0015	4.850	0	1 (0.2)
	**(h) Class 2 Excitable**					
**Model**	**No. Params**	**Inference Memory (MB)**	**Inference Time (s)**	**RMSE**	**VRD**	**F1-Score (Maximum Tolerance (ms))**
Izhikevich	/	3.04	0.031	0	0	1 (0)
SRM	19,534	0.86	0.068	1.645	0	1 (0.1)
SSRM	39,998	0.58	0.259	0.0002	0	1 (0)
RSRM	201	0.02	0.0008	10.298	668.784	0.768 (21.1)
RSSRM	201	0	0.0014	5.709	31.606	0.938 (0.3)
PCR	201	0.18	0.0007	10.438	1.19 × 10${}^{6}$	0.0713 (9.5)
RCR	201	0.81	0.0015	7.113	1461.463	0.19 (36)
	**(i) Spike Latency**					
**Model**	**No. Params**	**Inference Memory (MB)**	**Inference Time (s)**	**RMSE**	**VRD**	**F1-Score (Maximum Tolerance (ms))**
Izhikevich	/	3.12	0.031	0	0	1 (0)
SRM	10,001	0.97	0.047	0.044	0	1 (0)
SSRM	39,998	0.06	0.26	1.37	0	1 (0)
RSRM	102	0.02	0.0009	2.651	8091	0.024 (0)
RSSRM	102	0	0.0039	1.860	0	1 (0.4)
PCR	102	0.9	0.0007	1.946	0	1 (1.4)
RCR	102	1.13	0.0012	1.937	0	1 (1.5)
	**(j) Subthreshold Oscillations**					
**Model**	**No. Params**	**Inference Memory (MB)**	**Inference Time (s)**	**RMSE**	**VRD**	**F1-Score (Maximum Tolerance (ms))**
Izhikevich	/	2.36	0.024	0	0	1 (0)
SRM	10,001	0.52	0.034	0.228	0	1 (0)
SSRM	30,198	0.91	0.143	4.054	0	1 (0.41)
RSRM	97	0.01	0.0005	1.753	557.530	0.087 (0)
RSSRM	96	0	0.0006	4.053	0	1 (0.43)
PCR	97	0.72	0.0006	1.995	0	1 (0.23)
RCR	97	0.88	0.0003	4.054	0	1 (0.41)
	**(k) Resonator**					
**Model**	**No. Params**	**Inference Memory (MB)**	**Inference Time (s)**	**RMSE**	**VRD**	**F1-Score (Maximum Tolerance (ms))**
Izhikevich	/	0.63	0.005	0	0	1 (0)
SRM	1043	0.87	0.004	0.382	0	1 (0)
SSRM	4730	0.4	0.004	2.292	0	1 (11)
RSRM	10	0	0.0001	3.687	182	0.143 (0)
RSSRM	9	0	0.0003	2.308	5.057	0.5 (12.5)
PCR	10	0.95	0.0001	3.548	0	1 (1)
RCR	10	0.58	0.0001	2.299	11.378	0.4 (1)
	**(l) Integrator**					
**Model**	**No. Params**	**Inference Memory (MB)**	**Inference Time (s)**	**RMSE**	**VRD**	**F1-Score (Maximum Tolerance (ms))**
Izhikevich	/	1.03	0.008	0	0	1 (0)
SRM	2001	0.52	0.003	3.57	0	1.000 (0)
SSRM	10,598	0.56	0.017	3.16 × 10${}^{-7}$	0	1 (0)
RSRM	20	0.01	0.0001	11.655	7.46 × 10${}^{4}$	0.008 (0.23)
RSSRM	20	0	0.0004	9.171	0	1 (0.34)
PCR	20	0.17	0.0001	4.394	0	1 (0.06)
RCR	20	0.13	0.0001	9.205	0	1 (0.06)
	**(m) Rebound Spike**					
**Model**	**No. Params**	**Inference Memory (MB)**	**Inference Time (s)**	**RMSE**	**VRD**	**F1-Score (Maximum Tolerance (ms))**
Izhikevich	/	0.71	0.01	0	0	1 (0)
SRM	2159	1.04	0.002	0.147	0	1 (0)
SSRM	6998	0.95	0.007	0.928	0	1 (0)
RSRM	22	0.03	0.0001	12.432	2.88 × 10${}^{5}$	0.004 (1)
RSSRM	22	0	0.0001	6.171	0	1 (0.51)
PCR	22	1.67	0.0004	5.616	0	1 (0.3)
RCR	22	0.27	0.0001	6.613	0	1 (0.64)
	**(n) Rebound Burst**					
**Model**	**No. Params**	**Inference Memory (MB)**	**Inference Time (s)**	**RMSE**	**VRD**	**F1-Score (Maximum Tolerance (ms))**
Izhikevich	/	2.09	0.031	0	0	1 (0)
SRM	10,001	0.86	0.063	0.009	0	1 (0)
SSRM	49,998	0.58	0.405	5.963	0	1 (0.2)
RSRM	105	0.01	0.0008	5.963	4.22 × 10${}^{5}$	0.049 (6.9)
RSSRM	105	0	0.001	5.977	0	1 (0.2)
PCR	105	0.8	0.0008	4.039	2.44 × 10${}^{4}$	0.191 (1.8)
RCR	105	0.35	0.0018	5.963	0	1 (0.2)
	**(o) Threshold Variability**					
**Model**	**No. Params**	**Inference Memory (MB)**	**Inference Time (s)**	**RMSE**	**VRD**	**F1-Score (Maximum Tolerance (ms))**
Izhikevich	/	0.76	0.01	0	0	1 (0)
SRM	2946	1.06	0.005	4.233	0	1 (0)
SSRM	12,398	0.7	0.0242	6.512	0	1 (0.54)
RSRM	30	0	0.0002	8.765	3.7 × 10${}^{5}$	0.004 (0.2)
RSSRM	30	0	0.0002	6.521	0	1 (0.61)
PCR	30	0.16	0.0001	5.036	0	1 (0.31)
RCR	30	1.03	0.0002	6.518	0	1 (0.54)
	**(p) Bistability**					
**Model**	**No. params**	**Inference Memory (MB)**	**Inference Time (s)**	**RMSE**	**VRD**	**F1-Score (Maximum Tolerance (ms))**
Izhikevich	/	3.47	0.031	0	0	1 (0)
SRM	16,001	1.12	0.061	0.033	0	1 (0)
SSRM	39,998	0.24	0.25	4.37 × 10${}^{-9}$	0	1 (0)
RSRM	172	0	0.0008	19.727	365.366	0.154 (4.2)
RSSRM	172	0	0.0051	10.341	5.057	0.909 (0.4)
PCR	172	1.59	0.0016	15.401	728.203	0.49 (3.3)
RCR	172	1.05	0.0013	15.573	80.911	0.5 (7.2)
	**(q) Depolarizing After-potential**					
**Model**	**No. Params**	**Inference Memory (MB)**	**Inference Time (s)**	**RMSE**	**VRD**	**F1-Score (Maximum Tolerance (ms))**
Izhikevich	/	0.28	0.004	0	0	1 (0)
SRM	1001	0.86	0.001	0.09	0	1 (0)
SSRM	3290	0.16	0.002	6.34 × 10${}^{-7}$	0	1 (0)
RSRM	10	0.01	0.0005	18.203	2.34 × 10${}^{4}$	0.015 (0.88)
RSSRM	10	0	0.0005	10.802	0	1 (0.26)
PCR	10	0.18	0.0005	6.735	0	1 (0.22)
RCR	10	0.2	0.0001	10.269	1.264	0.667 (0.22)
	**(r) Accommodation**					
**Model**	**No. Params**	**Inference Memory (MB)**	**Inference Time (s)**	**RMSE**	**VRD**	**F1-Score (Maximum Tolerance (ms))**
Izhikevich	/	1.79	0.016	0	0	1 (0)
SRM	5103	0.84	0.014	0.06	0	1 (0)
SSRM	18,998	0.71	0.057	3.59 × 10${}^{-9}$	0	1 (0)
RSRM	51	0	0.0002	2.750	0	1 (10.3)
RSSRM	51	0	0.0003	2.438	0	1 (2.6)
PCR	51	1.46	0.0002	1.761	0	1 (0.3)
RCR	51	0.35	0.0003	3.050	0	1 (0.2)
	**(s) Inhibition-induced Spiking**					
**Model**	**No. Params**	**Inference Memory (MB)**	**Inference Time (s)**	**RMSE**	**VRD**	**F1-Score (Maximum Tolerance (ms))**
Izhikevich	/	3.35	0.031	0	0	1 (0)
SRM	16,001	0.6	0.062	0.004	0	1 (0)
SSRM	39,998	0.7	0.25	1.08 × 10${}^{-9}$	0	1 (0)
RSRM	160	0	0.0008	6.734	1.88 × 10${}^{4}$	0.013 (10.3)
RSSRM	160	0	0.0011	3.067	1.264	0.963 (0.9)
PCR	160	0.97	0.0007	5.495	7302	0.233 (16.2)
RCR	160	1.48	0.0012	5.237	0	1 (8.5)
	**(t) Inhibition-induced Bursting**					
**Model**	**No. Params**	**Inference Memory (MB)**	**Inference Time (s)**	**RMSE**	**VRD**	**F1-Score (Maximum Tolerance (ms))**
Izhikevich	/	3.21	0.031	0	0	1 (0)
SRM	16,001	0.86	0.065	0.007	0	1 (0)
SSRM	39,998	0.68	0.252	2.44 × 10${}^{-9}$	0	1 (0)
RSRM	156	0	0.0007	14.701	2.86 × 10${}^{7}$	0.035 (1.8)
RSSRM	155	0	0.0011	5.294	854	0.869 (3.2)
PCR	155	0.82	0.0007	9.400	1.31 × 10${}^{5}$	0.359 (8.5)
RCR	155	1.52	0.0014	11.965	0	1 (0.2)

^1^ Inference memory, the memory consumption during inference, of 0 MB indicates the actual memory usage is smaller than 10 KB. ^2^ VRD of 0 represents the actual VRD is tiny and is close to 0. Such a rounding error may come from Equation (21), which approximates the exact VRD involving kernels and integrals, and an error in numerical computations that is smaller than the machine epsilon. * They are pre-defined maximum tolerances considering biological plausibility and applicability.

**Table 3:** Average performance on twenty firing behaviors.

Model	No. Params	Inference Memory (MB) ^1^	Inference Time (s)	RMSE	VRD ^2^	F1-Score (Maximum Tolerance (ms))
Izhikevich	/	2.48	0.025	0	0	1 (0)
SRM	9895	0.83	0.043	0.587	0.063	0.999 (0.051)
SSRM	29,659	0.56	0.174	1.056	0	1 (1.056)
RSRM	100	0.02	0.0006	10.158	3.6 × 10${}^{6}$	0.123 (4.453)
RSSRM	100	0	0.0015	5.632	47.219	0.95 (1.444)
PCR	100	0.86	0.0006	7.265	9.5 × 10${}^{4}$	0.546 (11.19)
RCR	100	0.77	0.001	6.833	85.399	0.83 (7.049)

^1^ Inference memory, the memory consumption during inference, of 0 MB indicates the actual memory usage is smaller than 10 KB. ^2^ VRD of 0 represents the actual VRD is tiny and is close to 0. Such a rounding error may come from Equation (21), which approximates the exact VRD involving kernels and integrals, and an error in numerical computations that is smaller than the machine epsilon.

**Table 4:** Average performance on one-spike firing behaviors.

Model	No. Params	Inference Memory (MB) ^1^	Inference Time (s)	RMSE	VRD ^2^	F1-Score (Maximum Tolerance (ms))
Izhikevich	/	1.55	0.016	0	0	1 (0)
SRM	5028	0.83	0.018	0.982	0	1 (0)
SSRM	16,134	0.49	0.063	1.685	0	1 (1.328)
RSRM	50	0.02	0.0004	7.867	9.8 × 10${}^{4}$	0.144 (1.401)
RSSRM	50	0	0.0015	5.078	0.562	0.944 (1.989)
PCR	50	0.75	0.0004	4.330	0	1 (0.499)
RCR	50	0.63	0.0006	5.336	1.405	0.896 (0.57)

^1^ Inference memory, the memory consumption during inference, of 0 MB indicates the actual memory usage is smaller than 10 KB. ^2^ VRD of 0 represents the actual VRD is tiny and is close to 0. Such a rounding error may come from Equation (21), which approximates the exact VRD involving kernels and integrals, and an error in numerical computations that is smaller than the machine epsilon.

**Table 5:** Average performance on multiple-spike firing behaviors.

Model	No. Params	Inference Memory (MB) ^1^	Inference Time (s)	RMSE	VRD ^2^	F1-Score (Maximum Tolerance (ms))
Izhikevich	/	3.24	0.032	0	0	1 (0)
SRM	13,878	0.83	0.062	0.264	0.115	0.998 (0.093)
SSRM	40,725	0.62	0.265	0.542	0	1 (0.018)
RSRM	141	0.03	0.0008	12.032	6.5 × 10${}^{6}$	0.106 (6.95)
RSSRM	141	0	0.0014	6.086	85.394	0.954 (0.997)
PCR	141	0.95	0.0008	9.667	1.7 × 10${}^{5}$	0.175 (19.936)
RCR	141	0.87	0.0013	8.057	154.123	0.776 (12.35)

^1^ Inference memory, the memory consumption during inference, of 0 MB indicates the actual memory usage is smaller than 10 KB. ^2^ VRD of 0 represents the actual VRD is tiny and is close to 0. Such a rounding error may come from Equation (21), which approximates the exact VRD involving kernels and integrals, and an error in numerical computations that is smaller than the machine epsilon.

**Table 6:** Ranked Performance on twenty firing behaviors.

	RMSE				VRD				F1-Score			
	**RSRM**	**RSSRM**	**PCR**	**RCR**	**RSRM**	**RSSRM**	**PCR**	**RCR**	**RSRM**	**RSSRM**	**PCR**	**RCR**
(a) Tonic spiking	4	1	3	2	4	1	3	2	4	1	3	2
(b) Phasic Spiking	4	1	3	2	4	1	1	1	4	1	3	2
(c) Tonic Bursting	4	1	3	2	4	1	3	2	4	1	3	2
(d) Phasic Bursting	4	1	3	2	4	2	3	1	4	1	3	2
(e) Mixed Mode	4	2	3	1	4	1	3	2	4	1	3	2
(f) Spike Frequency Adaptation	4	1	3	2	4	1	3	2	4	1	3	2
(g) Class 1 Excitable	4	1	3	2	3	1	4	1	3	2	4	1
(h) Class 2 Excitable	3	1	4	2	2	1	4	3	2	1	4	3
(i) Spike Latency	4	1	3	2	4	1	1	1	4	1	2	3
(j) Subthreshold Oscillations	1	3	2	4	4	1	1	1	4	3	1	2
(k) Resonator	4	2	3	1	4	2	1	3	4	2	1	3
(l) Integrator	4	2	1	3	4	1	1	1	4	3	1	1
(m) Rebound Spike	4	2	1	3	4	1	1	1	4	2	1	3
(n) Rebound Burst	4	3	1	2	4	1	3	1	4	1	3	1
(o) Threshold Variability	4	3	1	2	4	1	1	1	4	3	1	2
(p) Bistability	4	1	2	3	3	1	4	2	4	1	3	2
(q) Depolarizing After-potential	4	3	1	2	4	1	1	2	4	2	1	3
(r) Accommodation	3	2	1	4	1	1	1	1	4	3	2	1
(s) Inhibition-induced Spiking	4	1	3	2	4	2	3	1	4	2	3	1
(t) Inhibition-induced Bursting	4	1	2	3	4	2	3	1	4	2	3	1
Mean	3.75	1.65	2.30	2.30	3.65	1.20	2.25	1.50	3.85	1.70	2.40	1.95

## Data Availability

Not applicable.

## Abbreviations

The following abbreviations are used in this manuscript:

SRM	Spike Response Model
RSRM	Regularized Spike Response Model
SSRM	Spectral Spike Response Model
RSSRM	Regularized Spectral Spike Response Model
PCR	Principal Component Regression
RCR	Raised Cosine Regression
RMSE	Root Mean Square Error
VRD	van Rossum Distance
